# Prevention of Bacterial Infection in Biliary and Pancreatic Endoscopy—A Review

**DOI:** 10.3390/diagnostics14242806

**Published:** 2024-12-13

**Authors:** Michał Kowalski, Adam Przybyłkowski

**Affiliations:** Department of Gastroenterology and Internal Medicine, Medical University of Warsaw, 02-097 Warsaw, Poland; michal.kowalski@uckwum.pl

**Keywords:** endoscopic retrograde cholangiopancreatography, sepsis, antibiotic prophylaxis, cholangitis, infections, adult, pediatric

## Abstract

Background: Endoscopic retrograde cholangiopancreatography (ERCP) is a key therapeutic procedure in diseases of the pancreas or bile ducts. The understanding and effective management of the risks associated with the procedure, especially in the context of possible infectious complications, is crucial for patients’ safety. The aim of this review was to analyze the results of studies on antibiotic prophylaxis for infectious complications of ERCP, pancreatoscopy, and cholangioscopy. Methods: This study is a review of the articles available in PubMed, Medline, and Embase published in the last 30 years. Results: Nineteen studies and six sets of guidelines on antibiotic prophylaxis before ERCP were retrieved. Conclusions: Based on the available studies and recommendations, it can be concluded that antibiotic prophylaxis before ERCP is beneficial for immunocompromised patients or those at risk of bacterial endocarditis. In other groups of patients, antibiotic prophylaxis reduces the risk post-ERCP bacteremia but does not significantly reduce the risk of cholangitis and infectious complications. The effectiveness of antibiotic prophylaxis in patients at risk of incomplete biliary drainage needs to be verified in further studies.

## 1. Introduction

Endoscopic retrograde cholangiopancreatography (ERCP) is an advanced endoscopic procedure the aim of which is to treat diseases of the bile ducts or pancreatic ducts. The incidence of complications after ERCP ranges from 1% to around 19–20% [1,2,3]. The most common are cholangitis and acute pancreatitis, which occur in 3% and 5.5% of patients, respectively [3]. Other, rare complications are bleeding-, perforation-, and sedation-related. Infections after ERCP are a consequence of the bacterial contamination of physiologically sterile bile and the subsequent translocation of bacteria into the main bloodstream. In healthy humans, there are mechanisms that protect bile from bacterial contamination. These include sphincter of Oddi tension, which is a gate keeper that prevents the entry of bacteria into the intestinal lumen, and tight junctions between hepatocytes, which prevent the entry of bacteria from bile into the bloodstream [1]. Additional mechanisms include the secretion of immunoglobulin IgA, which regulates the immune response and the antiseptic properties of bile salts, into bile. An equally important mechanism in preventing bile contamination is the normal flow of bile in the bile ducts [1,4]. There are two main routes by which bacteria translocate into the bile: blood-borne [1,3,5] and retrograde from the intestinal lumen [1,6]. In the first scenario, bacteria from the gastrointestinal tract migrate through the intestinal wall into the mesenteric veins and further portal vein to the liver and are then transported into the sinuses of the liver and the Disse space and directly into the bile [1,7]. In the case of the retrograde route, bacteria migrate via the papilla of Vater from the duodenum into the common bile duct, directly into the biliary tree, and cause bacteriobilia [1,8]. The ascending route is more prevalent for the bacterial colonization of the bile and biliary tree than the blood-borne route (Table 1). Bacteriobilia can lead to clinically significant bacteremia and therefore septic complications. Transient bacteremia itself does not trigger infection and septic complications in every case. Everyday activities such as tooth brushing (20% to 60%) or flossing (20–40%) are also associated with the occurrence of transient bacteremia [9,10,11]. The risk of clinically significant bacteremia is related to the type of biliary pathology and comorbidities [1]. In patients with malignant lesions, bacteremia is observed in 35% of cases; in patients with bile duct stones, in 70–80% of cases; and in 90% of patients with Vater’s papilla dysfunction. In ERCP patients who do not have biliary obstruction, the risk of bacteremia is approximately 6%, whereas for patients with risk factors, the estimated risk of bacteremia is 18% [10].

Mortality in severe post-ERCP cholangitis ranges from 10 to 16% [1]. In a study by Bilbao Mk. et al. covering 10,000 patients, mortality due to severe cholangitis was approximately 10% [16], while mortality in the pediatric population is relatively low [17].

The spectrum of bacteria that most commonly cause post-ERCP infectious complications mostly overlaps with the bacteria that cause transient bacteremia. These are Gram-negative bacteria of the groups *E. coli*, *Klebsiella* spp., and *Pseudomonas* spp., Gram-positive Enterococci species, anaerobic Bacteroides spp., and *Clostridium* spp. (Table 1) [5,9]. The aim of this review was to determine the actual indications for antibiotic prophylaxis prior to ERCP and to verify whether antibiotics prophylaxis has a real impact on the risk of complications after ERCP.

## 2. Methods

The PubMed (National Library of Medicine, Bethesda, MD, USA), Medline, and Embase databases were searched using the following keywords: ERCP, cholangioscopy, pancreatoscopy, sepsis, cholangitis, infection, antibiotic(s), prophylaxis, adult, pediatric. For the purposes of this review, randomized controlled trials [RCTs], retrospective studies, guidelines, and scientific society recommendations from 1994 to October 2024 were selected.

## 3. Results

**Figure 1:**
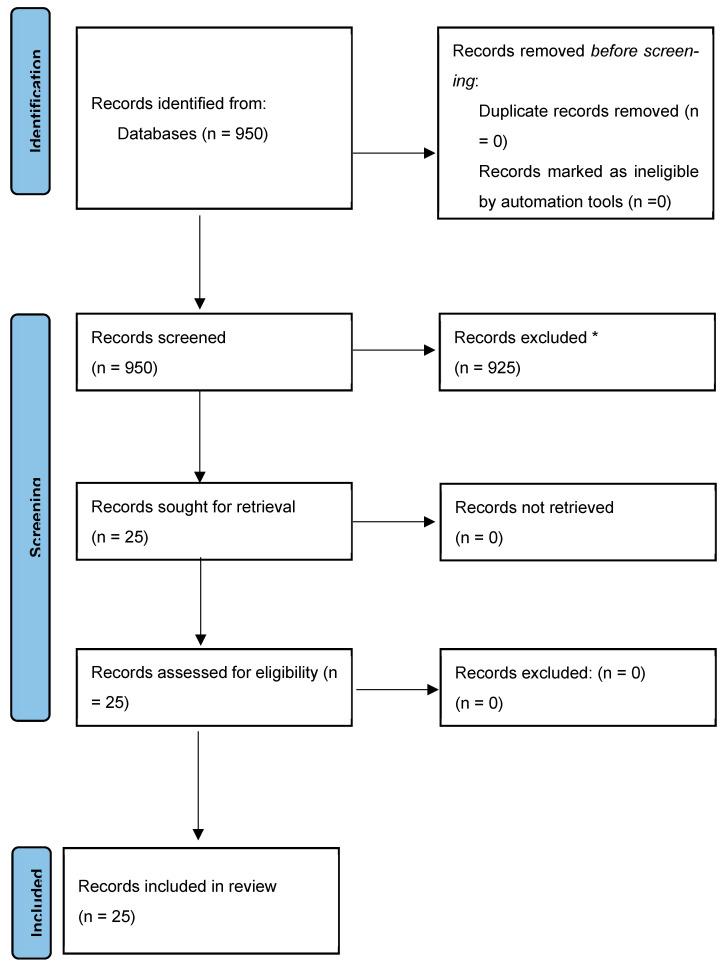
Infection prevention in endoscopic biliary and pancreatic procedures (PRISMA). * Reasons for excluding records or full-text articles were as follows: articles not dealing with antibiotic prophylaxis in ERCP, reviews, articles in a language other than English, single case reports.

An initial search identified 950 studies, 25 of which met the criteria for this narrative review. The PRISMA plot is presented in Figure 1.

Based on the search strategy, 25 publications were identified, including 16 RCTs, 3 retrospective studies, and 6 sets of guidelines/recommendations.

### 3.1. RCTs and Retrospective Studies

Among the twenty-five publications, two studies— a randomized study by Norouzi et al. [18] and a retrospective study by Gustaffson [19]—covered homogenous groups of patients, while the other concerned heterogenous groups of indications for ERCP (Table 2). Norouzi et al. [18] examined the effect of gentamicin added to the contrast medium on the rate of post-ERCP cholangitis in patients with non-calculous obstructive jaundice. There was no significant difference in the incidence of cholangitis between the group receiving gentamicin versus the group without antibiotics. Niederau et al. evaluated the effectiveness of 2 g cefotaxime versus no antibiotic [20]. They found that bacteremia and cholangitis occurred in eight patients and only in the group without prophylaxis (in four cases, ERCP had failed to decompress the biliary system completely). Ratanachu et al. tried to determine the influence of ciprofloxacin on cholangitis rates in cholestatic patients with adequate biliary drainage [21]. Of 48 patients who received 200 mg ciprofloxacin iv before ERCP, 24 continued it for the next 48 h after the procedure. The continual use of ciprofloxacin in patients with cholestasis after adequate biliary drainage procedures had no effect on cholangitis reduction. Finkelstein et al. evaluated the effectiveness of 1 g cefonicid versus no antibiotic and found that the rates of bacteriemia and cholangitis were similar in both groups [22]. Lorenz et al. prospectively assessed the effectiveness of a single prophylactic dose of 1500 mg iv cefuroxime [23]. The septicemia rate was 6.1% in the antibiotic group versus 10% in the group without an antibiotic. Similarly, Raty et al. evaluated the effectiveness of a single dose of 2 g of ceftazidime iv. before ERCP and found that a single dose of the antibiotic reduced the risk of cholangitis after the procedure (0 patients with cholangitis in antibiotic group versus 7 in the control group) [24]. Similar conclusions were reached in a study by Leem et al., who compared the administration of 1 g intravenous cefoxitin versus placebo [25]. Bacteremia was found in four patients in the antibiotic group versus eleven in the placebo group, and cholangitis in three patients in the antibiotic group versus eleven in the placebo. They concluded that antibiotic prophylaxis before ERCP in patients with biliary obstruction resulted in a significantly lower risk of infectious complications, especially cholangitis. In contrast, Spicak et al. did not confirm that the use of antibiotic prophylaxis before therapeutic ERCP reduced the risk of cholangitis or affected bacteremia [26]. Llach et al., who examined 600 mg of clindamycin and 80 mg of gentamycin, both given intramuscularly before ERCP, obtained similar results [27]. These authors stated that the prophylactic administration of clindamycin plus gentamycin reduced the incidence of neither bacteremia nor cholangitis. Smith et al. evaluated the efficacy of a 3 day course of oral amoxicillin with clavulanic acid after a single dose of ticarcillin with clavulanic acid in the prevention of ERCP-related sepsis [28]. Sepsis occurred in 10% of patients with a single dose of the antibiotic before ERCP and in 3% of individuals in the group that received the antibiotic for the additional 3 days. The study’s conclusion was that the performance of sphincterotomy and the presence of stones in the common bile duct were significant risk factors for infectious complications, and the administration of an additional dose of antibiotic may be justified in these cases. In a study by Hazel et al., patients with bile duct stones or biliary stenosis received piperacillin as prophylaxis [29]. There was no reduction in infections despite antibiotic administration. In a randomized, double-blind study, Baudouin et al. showed that piperacillin with tazobactam at a dose of 4 g 30 min before ERCP [30] reduced the infectious complication rate in patients with suspected biliary obstruction.

Three studies compared the effectiveness of two different antibiotics for prophylaxis. Mehal I et al. compared the efficacy of oral ciprofloxacin and intravenous cefuroxime [31]. The administration of ciprofloxacin was found to be as effective in the prevention of infectious complications after ERCP and was cheaper than cefuroxime. Davis et al. compared the efficacy of periprocedural oral 750 mg ciprofloxacin with 1 g iv. cefazolin in the prevention of septic complications in a group of patients with gallstones and in individuals with benign or malignant strictures [32]. There were no significant differences in the efficacy of the two antibiotics. Kim et al. compared the effectiveness of 400 mg intravenous moxifloxacin to 2 g ceftriaxone administered 90 min before ERCP [33]. The frequency of cholangitis and septicemia was comparable in both groups.

Thompson et al. retrospectively analyzed the effect of biliary pathology and antibiotic prophylaxis on the risk of cholangitis after ERCP [34]. In a group of ninety patients who had not received prophylaxis, cholangitis only occurred in three patients.

In a retrospective study, Gustaffson et al. analyzed a homogeneous group of 2144 patients with primary sclerosing cholangitis (PSC) treated with ERCP. A total of 1407 patients received antibiotic prophylaxis and 737 patients did not. The infection rate was 3.3% in the antibiotic group in comparison to 4.5% in the control group [19]. Pohl et al. examined the efficacy of ciprofloxacin in patients with PSC and biliary stenosis treated with ERCP [35]. Bacteria were found in the bile of ten out of twelve patients; therefore, it was concluded that short-term antibiotic treatment was not effective in bacteria eradication from the biliary tract.

Olsson et al. performed a registry study based on the Swedish Registry of Gallstone Surgery and ERCP (covering more than 22,000 ERCP procedures). The use of antibiotic prophylaxis reduced the number of septic complications by 15%. It also reduced the risk of overall post-ERCP adverse events by 24%, especially in patients with mechanical jaundice and stones in the bile ducts [36].

In the aforementioned study by Gustaffson et al., 3.4% of patients on antibiotic prophylaxis had an infection after cholangioscopy, versus 3.7% in the group without an antibiotic [19]. In a retrospective study, Turowski et al. evaluated 206 patients examined with cholangioscopy [37]. In their study, cholangitis occurred in 1% of patients who received antibiotics versus 12.8% in the group without antibiotics.

No studies on antibiotic prophylaxis in pancreatoscopy were found. Similarly, no studies on antibiotic prophylaxis before ERCP, cholangioscopy, or pancreatoscopy in children were retrieved.

**Table 2:** Studies on antibiotic prophylaxis before pancreatobiliary endoscopy.

Author	Type of Study	Indications	Intervention	Bacteremia (*n*/*N*) *	Cholangitis (*n*/*N*)	Authors’ Conclusion
Niederau et al., 1994 [20]	RCT	diagnostic or therapeutic ERCP	cefotaxime 2 g iv. 15 min before ERCP	antibiotic: 0/50 control: 4/50	antibiotic: 0/50 control: 4/50	Cefotaxime can reduce the incidence of bacteremia and sepsis.
Byl et al., 1995 [30]	RCT	diagnostic or therapeutic ERCP	piperacillin 4 g iv.	antibiotic: 2/34 control: 7/34	antibiotic: 2/34 control:5/34	Antimicrobial prophylaxis significantly reduces the incidence of septic complications after therapeutic ERCP among patients presenting with cholestasis.
Mehal et al., 1995 [31]	RCT	radiological evidence of biliary obstruction	1.ciprofloxacin 750 mg po. 2.cefuroxime 1.5 g iv.	antibiotic 1:1/100 antibiotic 2:1/100	antibiotic 1:1/100 antibiotic 2:1/100	A pre- and post-ERCP oral ciprofloxacin regime is safe and provides effective prophylaxis against ERCP-induced cholangitis and septicemia in high-risk patients. It is also more economical than a regime of intravenous cefuroxime and does not require nursing staff with training in intravenous techniques.
Davis et al., 1998 [32]	RCT	radiological evidence of biliary obstruction	1.ciprofloxacin po 750 mg 2.cephazolin 1 g iv.	antibiotic: 10/77 antibiotic 2: 2/72	antibiotic 1: 0/77 antibiotic 2: 3/72	Oral ciprofloxacin is a cost-effective prophylactic agent for high-risk ERCP.
Ratanachu et al., 2007 [21]	RCT	therapeutic ERCP	ciprofloxacin 200 mg iv.	not covered	antibiotic: 1/22 control: 2/26	Continual use of ciprofloxacin in patients with cholestasis after adequate biliary drainage procedures plays no role in reducing cholangitis.
Finkelstein et al., 1996 [22]	RCT	diagnostic or therapeutic ERCP	cefonicid 1 g iv.	antibiotic: 3/88 control: 2/91	antibiotic: 7/88 control: 2/91	Infectious complications could not be prevented by cefonicid prophylaxis.
Lorenz at al., 1996 [23]	RCT	therapeutic eRCP	cefuroxime 1.5 g iv.	antibiotic: 3/49 control: 8/50	not covered	The differences obtained between the two groups were not statistically different.
Van del Hazel et al., 1996 [29]	RCT	diagnostic or therapeutic ERCP	piperacillin 4 g iv.	not covered	antibiotic: 12/270 control: 17/281	Single-dose prophylaxis with piperacillin is not associated with a clinically significant reduction in the incidence of acute cholangitis after ERCP.
Raty et al., 2001 [24]	RCT	diagnostic or therapeutic ERCP	ceftazidime 2 g iv.	not covered	antibiotic: 0/155 control 7/160	Antibiotic prophylaxis effectively decreases the risk of cholangitis after ERCP.
Leem et al., 2024 [25]	RCT	biliary obstruction	cefoxitin 1 g iv.	antibiotic: 4/176 control: 11/176	antibiotic: 3/76 control: 11/173	Antibiotic prophylaxis before ERCP in patients with biliary obstruction resulted in a significantly lower risk of infectious complications, especially cholangitis, than placebo.
Spicak et al., 2002 [26]	RCT	biliary obstruction	amoxicillin with clavulanic acid 2.4 g iv.	antibiotic: 18/77 control: 24/88	antibiotic: 4/77 control: 3/88	Antibiotic therapy before therapeutic ERCP does not reduce the risk of complicating cholangitis and does not influence bacteremia.
Llach et al., 2006 [27]	RCT	diagnostic or therapeutic ERCP	clindamycin 600 mg and gentamicin 80 mg im.	antibiotic: 2/31 control: 2/30	antibiotic: 1/31 control 1/30	Clindamycin plus gentamicin does not reduce the incidence of bacteremia and cholangitis.
Sciume et al., 2004 [38]	RCT	ERCP with state implantation due to biliary obstruction	levofloxacin 500 mg	not covered	not covered	In patients in group 1, “stent patency in situ” was 50% longer than in group 2, with a lower incidence of cholangitis and hospital admittance.
Norouzi et al., 2013 [18]	RCT	non-calculous obstructive jaundice	gentamicin added to contrast during ERCP	not covered	antibiotic: 5/57 control: 5/57	Adding gentamicin to contrast media had no significant effect on the incidence of post-ERCP cholangitis.
Kim et al., 2017 [33]	RCT	biliary obstruction	moxifloxacin 400 mg iv or ceftriaxone 2 g iv	antibiotic 1:1/43 antibiotic 2: 2/43	antibiotic 1: 1/43 antibiotic 2: 1/43	Intravenous moxifloxacin is not inferior to intravenous ceftriaxone for the prophylactic treatment of post-ERCP cholangitis and cholangitis-associated morbidity.
Smith et al., 1996 [28]	RCT	diagnostic or therapeutic ERCP	ticarcillin 1.6 g iv with clavulanic acid 1 h before ERCP or ticarcillin with clavulanic acid before ERCP and 3 days’ oral amoxycillin and clavulanic acid 1 g thereafter	not covered	not covered	Three days of oral amoxicillin and clavulanic acid after a single dose of intravenous antibiotics is recommended in patients at an increased risk of complications.
Gustafsson et al., 2023 [19]	Retrospective	primary sclerosing cholangitis	various antibiotics	not covered	not covered	Patients with PSC who undergo ERCP have the same frequency of adverse events regardless of whether antibiotics were used.
Thompson et al., 2002 [34]	Retrospective	diagnostic or therapeutic ERCP	various antibiotics	not covered	not covered	Single-dose antibiotic prophylaxis in the setting of presumed biliary obstruction to prevent ERCP-related cholangitis is a cost-saving strategy.
Olsson et al., 2015 [36]	Retrospective	diagnostic or therapeutic ERCP	various antibiotics	not covered	not covered	The use of prophylactic antibiotic therapy before ERCP for the three main indications of gallstones, tumors, and mechanical jaundice reduces the risk of septic complications by 15%.

* *n*—numbers of patients with specified event; *N*—number of patients in the study group.

### 3.2. Guidelines and Recommendations

Six publications covering recommendations and guidelines were retrieved (Table 3). All societies recommend antibiotic prophylaxis before ERCP in patients at risk of incomplete biliary drainage. In the recommendations of five societies, antibiotic prophylaxis is advised for patients with PSC, malignancy, neutropenia (defined as <500 neutrophils/µL), and in patients after liver transplantation. According to four societies, advanced hematologic tumors are also an indication for antibiotic prophylaxis. Only three societies recommend prophylaxis in patients with pancreatic pseudocysts and in patients with synthetic vascular grafts (within 6 months after implantation).

All societies do not recommend the use of antibiotic prophylaxis in patients without risk factors in whom the achievement of complete biliary drainage is expected.

The ESPHGAN guidelines [43] and ESGE [43] guidelines do not cover antibiotic prophylaxis before ERCP in the pediatric population.

## 4. Discussion

In the latest guidelines of the American and British Gastroenterological Societies, endoscopic procedures at high risk for bacteremia, which include ERCP in selected conditions, are indications for antibiotic prophylaxis. The use of antibiotic prophylaxis prior to ERCP is largely dependent on the individual patient’s clinical situation, concomitant burdens, and in-house experience. Antibiotic prophylaxis is recommended for patients qualified for ERCP and belonging to high-risk groups. High-risk groups include patients with risk of incomplete biliary drainage, cirrhosis with ascites/gastrointestinal bleeding, liver transplant recipients, and patients with neutropenia or with vascular grafts within 6 months of implantation, as well as patients at risk of bacterial endocarditis [5,10]. For patients who do not belong to the aforementioned risk groups, the use of antibiotics is debatable and is claimed to not be associated with a reduction in infection risk.

In 1988, Siegman-Igra et al. published a retrospective study on 720 ERCPs and proved that the use of antibiotics was associated with a reduction in infectious complications from 4.3% to 0%; however, in about 60% of the analyzed cases, the reason for ERCP was not specified, and neither were data on the type of antibiotic prophylaxis used [30].

A review by Brand et al. reached the conclusion that prophylactic antibiotics reduce bacteriaemia and seem to prevent cholangitis and septicemia in patients treated with elective ERCP [44].

In a meta-analysis by Merchan et al., who compared the results from 10 randomized trials on antibiotic prophylaxis in ERCP involving a group of 1757 patients, prophylactic antibiotic use reduced the risk of bacteremia but did not reduce the risk of infectious complications [3]. The authors confirmed that the risk of complications such as cholangitis or sepsis after ERCP is mainly related to incomplete biliary drainage and that full drainage should be sought first.

Similar results were found by Allison et al., who prepared the guidelines on behalf of the endoscopy committee of the BSG [9]. Olsson et al. studied a national population-based study cohort (registered in the Swedish Registry of Gallstone Surgery and ERCP) and analyzed 22,000 ERCPs with the three most common indications for the procedure: cholelithiasis, mechanical jaundice, and tumor [36]. They found that the use of antibiotic prophylaxis in patients with gallstones reduces the risk of adverse events after the procedure by 23%. However, the scope of their cohort study did not cover the patients’ comorbidities; therefore, Olsson et al.’s results cannot be translated into universal guidelines [9].

In a review article by Subhani et al., who summarized the results of available randomized clinical trials on the efficacy of individual antibiotics in the prevention of infectious complications after ERCP, the conclusion was made that antibiotic prophylaxis before ERCP is recommended for patients at high risk of infective endocarditis, while for patients with incomplete biliary drainage, antibiotics should be started immediately after ERCP and continued until complete drainage is achieved [1].

Based on the available research results, it may be concluded that antibiotic prophylaxis should be used in patients at risk of incomplete biliary drainage. However, there is no clear definition of which precisely defined conditions the risk of incomplete drainage refers to. It may be assumed that patients with PSC, hilar tumors, retained bile stones, multiple liver metastases, or those with a history of recurrent cholangitis could form a group at high risk of incomplete biliary drainage. The completeness of biliary drainage can only be reliably assessed after ERCP; therefore, some patients who should receive prophylaxis before ERCP receive it just after the procedure. This mode of antibiotic administration cannot be equated with prophylaxis. According to the World Health Organization’s (WHO) Surgical Safety Checklist, antimicrobial prophylaxis should be given 60 min or less before the skin incision (which in the case of ERCP corresponds to duodenal papilla instrumentation) to achieve adequate antibiotic tissue concentration.

Only individual studies have evaluated indications for antibiotic prophylaxis in homogeneous groups of patients qualified for ERCP; moreover, the endpoints of the published results vary. Based on the retrieved publications, it was not possible to prove a clear advantage of antibiotic administration in the prevention of infectious complications after ERCP or ERCP with subsequent cholangioscopy or pancreatoscopy.

We did not find studies on antibiotic prophylaxis in pediatric patients. In a meta-analysis by Danielle et al., with a clinical database of more than 2600 patients, the authors found that the most common post-ERCP complications in the pediatric population are acute pancreatitis (4.7%), bleeding (0.6–1%), and infection (0.8%) [45]. Similar conclusions were reached in a study by Gomez et al. that analyzed case series of 30 children who underwent 65 ERCP procedures. The results showed that septic complications in the pediatric population are relatively rare (1.5%). In addition, the mortality rate was 0% in all studies reviewed. These results suggest that infectious complications after ERCP in the pediatric population are very rare [17].

Data on antibiotic prophylaxis before pancreatoscopy are too weak to draw reliable conclusions. Further studies on antibiotic prophylaxis efficacy in the population of patients at risk of incomplete biliary drainage are needed. Before the completion of such well-designed randomized clinical studies, it is not possible to make reliable grade A recommendations.

## 5. Conclusions

Based on expert opinions and the available clinical experience, antibiotic prophylaxis before ERCP, cholangioscopy, or pancreatoscopy is recommended for patients with risk factors for bacterial endocarditis, cirrhotic patients with ascites/gastrointestinal bleeding, liver transplant recipients, and patients with neutropenia or with vascular grafts within 6 months of implantation. In patients not belonging to these risk groups, the administration of antibiotics before ERCP reduces the risk of bacteremia but does not affect the risk of cholangitis. In individuals with risk factors for incomplete drainage, such as retained gallstones, PSC, primary or secondary tumors of the liver, and IgG4-related sclerosing cholangitis, antibiotic prophylaxis seems to be a reasonable approach. Alternatively, initial treatment (which is not prophylaxis) of biliary infection can be started during ERCP if complete biliary drainage has not been achieved. The proposed strategies need to be verified in clinical studies.

**Figure 2:**
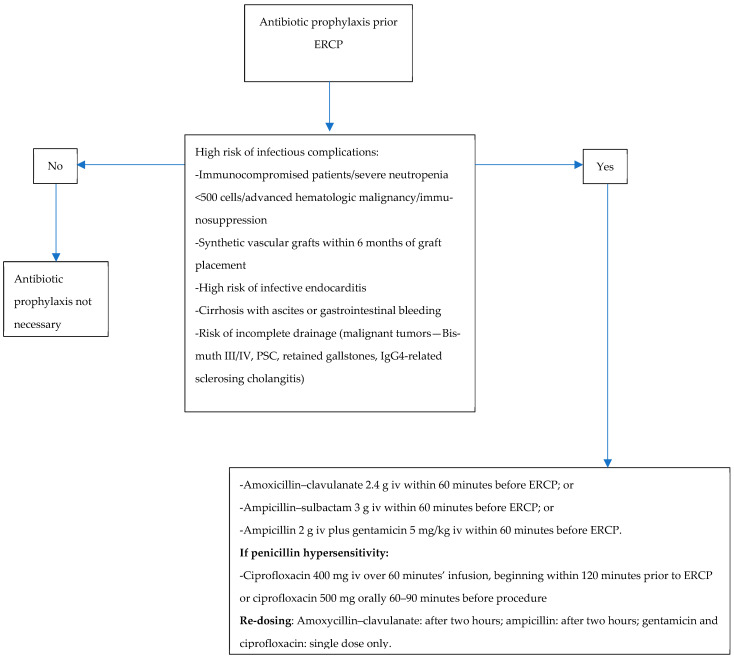
Antibiotic prophylaxis prior to ERCP.

The choice of antibiotic for prophylaxis should follow the local epidemiology of microbial resistance and should cover the spectrum of the most common Gram-negative bacteria from the Enterobacteriaceae found in the bile. The proposed antibiotic prophylaxis is presented in Figure 2. In line with the European Medicine Agency’s opinion published in “Quinolone and fluoroquinolone Article-31 referral outcome” (EMA/175398/2019), we do not recommend ciprofloxacin as a first line in prophylaxis due to the risk of potentially irreversible adverse drug reactions related to the use of fluoroquinolones, as well as the risk of Clostridioides difficile infection [46]. In case of incomplete biliary drainage, antibiotics used for prophylaxis should be continued for three days. If there is no evidence of cholangitis, antibiotics may be discontinued.

## Figures and Tables

**Table 1:** Prevalence of individual bacterial strains in bile according to available studies.

Clinical Condition	Number of Study Participants	Bacteria-Positive Bile Culture	Bacteria Strains							Reference
			*E. coli*	*Klebsiella* spp.	Other Enterobacteriacae	*Pseudomonas* spp.	*Bacteroides* spp.	*Enterococcus* spp.	Other Gram-Positive Bacteria	
CBD * stones and clear bile	230	76%	52%	14%	22%	0%	0.5%	13%	8%	[12]
CBD stones with turbid bile	315	92%	57%	18%	23%	0%	1%	11%	5%	[12]
Obstructive jaundice	134	66%	52%	35%	22%	25%	18%	40%	35%	[13]
CBD stones and 80% cholangitis	896	78%	52%	39%	23%	14%	6%	40%	32%	[14]
Biliary surgery	934	35%	58%	22%	9%	0,3%	5%	0%	21%	[15]

* CBD—common bile duct.

**Table 3:** Indications for antibiotic prophylaxis before biliary and pancreatic endoscopy according to guidelines and recommendations.

Clinical Condition	Society Name and Date of Guideline Issue					
	ASGE [10] 2015	PTGE [39] 2015	ESGE [40] 2022	BSG [9] 2009	CAG [41] 1999	KSGE [42] * 2013
Bile duct obstruction and complete drainage	no	no	no	no	no	no
Bile duct obstruction and incomplete drainage	yes	yes	yes	yes	yes	yes
Primary sclerosing cholangitis	yes	yes	yes	yes	not covered	yes
Malignancy	yes	yes	yes	yes	not covered	yes
Cholangiocarcinoma Bismuth III/IV	not covered	yes	not covered	yes	not covered	not covered
Caroli disease	not covered	yes	not covered	yes	not covered	not covered
Necrosis of the pancreas or pancreatic pseudocysts	not covered	yes	not covered	yes	not covered	yes
Prosthetic joints	no	not covered	not covered	not covered	no	not covered
Cirrhosis with GI bleeding	yes	not covered	not covered	not covered	not covered	yes
Neutropenia <500 cells/µl	yes	yes	yes	yes	yes	not covered
Advance hematologic malignancy	yes	yes	yes	yes	not covered	not covered
Immunosuppression	yes	not covered	yes	not covered	yes	not covered
Liver transplant recipients	yes	yes	yes	yes	yes	not covered
Synthetic vascular grafts or other nonvalvular cardiovascular devices	yes	no	not covered	yes	yes	no
Before cholangioscopy	not covered	not covered	yes	not covered	not covered	not covered

ASGE—American Society for Gastrointestinal Endoscopy; PTGE—Polish Society of Gastroenterology; ESGE—European Society of Gastrointestinal Endoscopy; BSG—British Society of Gastroenterology; CAG—Canadian Association of Gastroenterology; KSGE—Korean Society of Gastrointestinal Endoscopy; * was extracted from the official journal of the Korean Society of Gastrointestinal Endoscopy.

## Data Availability

All data are available in Pub-Med, Medline, and Embase.
